# Sex-Specific Differences in Patients With and Without Nickel Hypersensitivity Undergoing Patent Foramen Ovale Closure

**DOI:** 10.1016/j.jacadv.2025.102186

**Published:** 2025-09-01

**Authors:** Anastasios Apostolos, Stamatios Gregoriou, Maria Drakopoulou, Georgios Trantalis, Nikolaos Ktenopoulos, Konstantina Aggeli, Georgios Tsivgoulis, Alexander Stratigos, Konstantinos Tsioufis, Konstantinos Toutouzas

**Affiliations:** a Unit of Structural Heart Diseases, First Department of Cardiology, School of Medicine, National and Kapodistrian University of Athens, Hippocration General Hospital of Athens, Athens, Greece; b First Department of Cardiology, School of Medicine, National and Kapodistrian University of Athens, Hippocration General Hospital of Athens, Athens, Greece; c Royal Brompton and Harefield Hospitals, Guy's and St Thomas' NHS Foundation Trust, London, United Kingdom; d First Department of Dermatology and Venereology, School of Medicine, National and Kapodistrian University of Athens, Andreas Syggros Hospital, Athens, Greece; e Second Department of Neurology, School of Medicine, National and Kapodistrian University of Athens, Attikon University Hospital, Athens, Greece; f Department of Neurology, University of Tennessee Health Science Center, Memphis, Tennessee, USA

**Keywords:** allergy, foramen ovale, hypersensitivity, metal, nickel, patent, PFO

**Figure:**
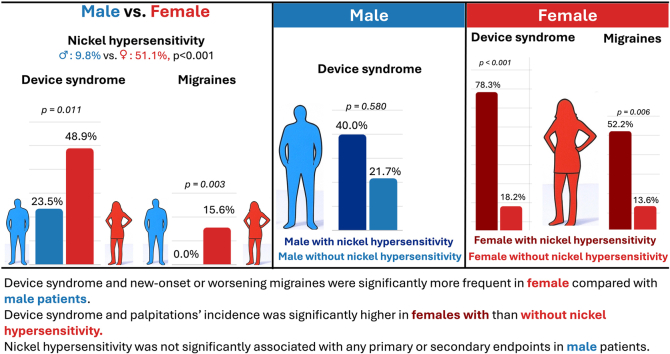

Transcatheter patent foramen ovale (PFO) closure has been a safe and effective therapeutic approach for secondary stroke prevention. The Amplatzer PFO Occluder (Abbott Laboratories) and the Gore Cardioform Septal Occluder (GSO) (W. L. Gore & Associates) are used for transcatheter PFO closure. Both devices are constructed from nitinol, an alloy of nickel and titanium. Nickel, a type IV hypersensitivity allergen, sensitizes about 20 to 30% of adults, with sex disparity: female patients exhibit 2–3x higher nickel allergy prevalence.^1^ However, the impact of systemic exposure to nickel following device placement is still being studied. Recently, Investigation of Nickel Sensitization After Percutaneous Implantation of Patent Foramen Ovale Occluder (INSPIRE) trial, a double-blinded, randomized study showed that the incidence of device syndrome, a composite of patient-reported symptoms was significantly higher in patients with nickel hypersensitivity compared with those without.^2^ However, it is unknown how sex-specific disparities in nickel hypersensitivity prevalence are also associated with adverse events. The aim of this secondary analysis is to evaluate whether preprocedural nickel hypersensitivity confers a sex-specific risk of adverse events following PFO closure.

**What is the clinical question being addressed?**

Does the preprocedural nickel hypersensitivity confer a sex-specific risk of adverse events following patent foramen ovale closure?

**What is the main finding?**

Our sex-stratified analysis revealed that the device syndrome and new-onset or worsening migraines were significantly more frequent in female than in male patients.

This is a sex-stratified, secondary analysis of the INSPIRE (NCT04713683).^2^ This analysis used sex assigned at birth as recorded in the trial. The study protocol, which adhered to the principles of the Declaration of Helsinki, was approved by the Institutional Review Boards of both participating hospitals and by the bioethics committee of Medical School of University of Athens. Written informed consent was obtained from all patients. All patients underwent patch testing prior the procedure following a blinded approach.

The primary endpoint of the analysis was “device syndrome”, a composite endpoint including at least one of the following: patient-reported new-onset chest pain, new-onset or worsening palpitations, new-onset or worsening migraines, dyspnea, or rash.^3^ The secondary endpoints included the composite outcomes of the primary endpoint, documented atrial arrhythmias, fever, major or minor bleeding, ischemic or hemorrhagic stroke, and all-cause mortality.

Continuous variables are reported as mean ± SD (normally distributed) or median (IQR) (non-normally distributed), assessed by Kolmogorov-Smirnov test. Group comparisons used Student’s I-test or Mann-Whitney U test for continuous variables, and chi-square or Fisher exact test for categorical variables (counts and percentages). Analyses followed intention-to-treat, with significance at *P* < 0.05, using Jamovi (v2.5).

Of the 96 patients of the main analysis, the majority were male (n = 51, 53.1%) and the rest were female patients (n = 45, 46.9%). Female patients were younger (median 43 [38-47] vs 49 [41-56] years; *P* = 0.009), had higher rates of autoimmune disorders (5/45 [11.1%] vs 0/51 [0%]; *P* = 0.024), and nickel hypersensitivity (23/45 [51.1%] vs 5/51 [9.8%]; *P* < 0.001). The incidence of device syndrome was significantly higher in female than in male patients (22 [48.9%] vs 12 [23.5%]; *P* = 0.011). The new-onset or worsening migraines, a component of device syndrome, (7 [15.6%] vs 0 [0.0%]; *P* = 0.003) were significantly more frequent in female patients. Secondary endpoints, including atrial arrhythmias (4/51 [7.8%] males vs 4/45 [8.9%] females; *P* = 1.000), fever (1/51 [2.0%] vs 1/45 [2.2%]; *P* = 1.000), minor bleeding (0/51 [0%] vs 1/45 [2.2%]; *P* = 0.469), and major bleeding, stroke, or mortality (0% in both groups), showed no significant sex differences. In male patients, nickel hypersensitivity was not significantly associated with any clinical endpoints, although limited by the small subgroup size. In female patients, device syndrome was observed in 18/23 (78.3%) of those with nickel hypersensitivity, compared with 4/22 (18.2%) of those without (*P* < 0.001). Palpitations occurred in 12/23 (52.2%) female patients with nickel hypersensitivity, significantly more than 3/22 (13.6%) without (*P* = 0.006) (Figure 1).

**Figure 1:**
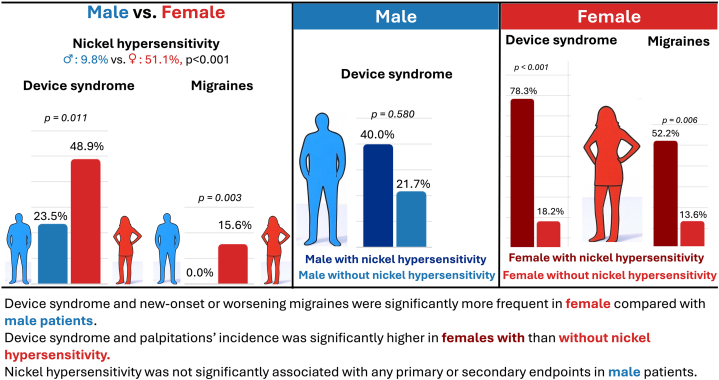
Sex-Specific Differences in Patients With and Without Nickel Hypersensitivity Undergoing Patent Foramen Ovale Closure

To the best of our knowledge, this is the first analysis to date investigating sex-stratified differences in patients with and without nickel hypersensitivity having undergone PFO closure. The main findings are 3-fold.

i. Device syndrome and the new-onset or worsening migraines were significantly more frequent in female compared with male patients.
ii. Device syndrome and palpitations’ incidence was significantly higher in female patients with than without nickel hypersensitivity.
iii. Nickel hypersensitivity was not significantly associated with any primary or secondary endpoints in male patients.

Interestingly, the majority of the patients with nickel hypersensitivity and severe allergic reaction in the literature are female. In a cohort of 58 patients having undergone surgical explantation of atrial septal defect occluders, due to nickel allergy, 95% of the reported cases were female.^4^ Consistent with prior studies, we observed a higher proportion of female patients with nickel hypersensitivity. Given the subjective nature of several components of device syndrome, the increased reporting by nickel-hypersensitive female compared to male patients could be partially attributed to a greater susceptibility to nocebo effects; however, this is unlikely to be present in our study as the skin patch testing was blinded. Alternatively, hormonal and genetic pathways might underlie this sex-specific vulnerability. Notably, female patients with endometriosis or atopic dermatitis have higher rates of nickel hypersensitivity, compared to those without. Rigatelli et al noted that all patients in their cohort were premenopausal women, and almost all were treated with oral contraceptives.^3^

Both the Amplatzer PFO Occluder and the GSO device are known to release nickel ions into the bloodstream, which is subsided within 90 days, coinciding with device endothelialization. An in vitro study demonstrated that the GSO device releases less nickel compared to the Amplatzer device, and therefore it might be safer for patients with nickel hypersensitivity. In accordance with our main analysis, which did not find any significant differences in outcomes between the 2 devices, this analysis did not show that device selection affects clinical outcomes in male and female patients, with and without nickel hypersensitivity.^2^

Nickel skin patch tests are the gold standard for diagnosing nickel hypersensitivity, but there is no recommendation for preprocedural nickel skin patch testing.^5^ Our study suggests that nickel skin patch testing before PFO closure might be most beneficial in female, considering that device syndrome did not differ between male patients with and without nickel hypersensitivity.

Our analysis has several limitations. First, this is relatively small cohort with a limited number of endpoints. This study was a post hoc analysis of a prospective, randomized study; therefore, our original sample was not powered to detect differences between the 2 sexes. Second, randomization was not stratified by sex, and the lower proportion of male patients suggests a cautious interpretation of the results given the chance of a type II error. Third, device syndrome includes multiple subjective symptoms and may be affected by nocebo effects, especially in those with nickel hypersensitivity.

Our study found that nickel hypersensitivity was more common in females and that device syndrome and the new-onset or worsening migraines were more frequent in females than in males. In female patients with nickel hypersensitivity, the incidence of device syndrome and palpitations was significantly higher than in those without nickel hypersensitivity.

## Funding support and author disclosures

This study has received an educational grant from Hellenic Society of Cardiology. The authors have reported that they have no relationships relevant to the contents of this paper to disclose.
